# The Effects of Hand Tremors on the Shooting Performance of Air Pistol Shooters with Different Skill Levels

**DOI:** 10.3390/s24082438

**Published:** 2024-04-11

**Authors:** Yu Liu, Nijia Hu, Mengzi Sun, Feng Qu, Xinglong Zhou

**Affiliations:** 1 Biomechanics Laboratory, School of Sport Science, Beijing Sport University, Beijing 100084, China; liuyu-1-2-3@163.com (Y.L.); qufeng929@163.com (F.Q.); 2 NeuroMuscular Research Center, Faculty of Sport and Health Sciences, University of Jyväskylä, FI-40014 Jyväskylä, Finland; hunijia@gmail.com; 3 School of Sports Science, Nanjing Normal University, Nanjing 210023, China; 12199@njnu.edu.cn

**Keywords:** physiological tremor, tremor amplitude, tremor complexity, multiscale entropy, muscle activation

## Abstract

Physiologic hand tremors are a critical factor affecting the aim of air pistol shooters. However, the extent of the effect of hand tremors on shooting performance is unclear. In this study, we aim to explore the relationship between hand tremors and shooting performance scores as well as investigate potential links between muscle activation and hand tremors. In this study, 17 male air pistol shooters from China’s national team and the Air Pistol Sports Center were divided into two groups: the elite group and the sub-elite group. Each participant completed 40 shots during the experiment, with shooters’ hand tremors recorded using three-axis digital accelerometers affixed to their right hands. Muscle activation was recorded using surface electromyography on the right anterior deltoid, posterior deltoid, biceps brachii (short head), triceps brachii (long head), flexor carpi radialis, and extensor carpi radialis. Our analysis revealed weak correlations between shooting scores and hand tremor amplitude in multiple directions (middle-lateral, ML: r^2^ = −0.22, *p* < 0.001; vertical, VT: r^2^ = −0.25, *p* < 0.001), as well as between shooting scores and hand tremor complexity (ML: r^2^ = −0.26, *p* < 0.001; VT: r^2^ = −0.28, *p* < 0.001), across all participants. Notably, weak correlations between shooting scores and hand tremor amplitude (ML: r^2^ = −0.27, *p* < 0.001; VT: r^2^ = −0.33, *p* < 0.001) and complexity (ML: r^2^ = −0.31, *p* < 0.001) were observed in the elite group but not in the sub-elite group. Moderate correlation were found between the biceps brachii (short head) RMS and hand tremor amplitude in the VT and ML directions (ML: r^2^ = 0.49, *p* = 0.010; VT: r^2^ = 0.44, *p* = 0.025) in all shooters, with a moderate correlation in the ML direction in elite shooters (ML: r^2^ = 0.49, *p* = 0.034). Our results suggest that hand tremors in air pistol shooters are associated with the skill of the shooters, and muscle activation of the biceps brachii (long head) might be a factor affecting hand tremors. By balancing the agonist and antagonist muscles of the shoulder joint, shooters might potentially reduce hand tremors and improve their shooting scores.

## 1. Introduction

Pistol stability is a key determinant of accuracy in air pistol shooting, and maintaining a stable posture while aiming is of critical importance [1,2,3,4,5]. Physiologic tremors, which are small involuntary oscillations of the limbs, can significantly impact shooting accuracy [6]. In air pistol shooting, minor deviations can lead to significant differences in shooting scores [3,7,8]. Therefore, minimizing hand tremors is essential for improving hand stability and improving scores [5]. However, effectively reducing tremors requires that we understand the characteristics of the tremors that occur during the aiming phase of air pistol shooting and their influencing factors [3].

Evaluating hand tremors involves analyzing accelerometer (ACC) signals in terms of both time domain and frequency [3,9]. Time domain analysis includes assessing tremor amplitude and complexity [9,10], with the root mean square (RMS) commonly used to quantify the amplitude. A low tremor amplitude is a prerequisite for elite shooters [5,6,9]. Some studies have demonstrated that elite air pistol shooters rely on body and arm stability for aiming [4]. However, other studies have suggested that for elite shooters, hand stability, rather than body stability, is the primary factor affecting shooting performance [9,11]. Two studies using principal component analysis identified aiming time, stability of hold, and aiming accuracy as three key technical components for achieving good scores in air pistol shooting [12,13]. The stability of the hold is largely dependent on the incidence of hand tremors. Thus, hand tremors may be a key factor affecting shooting scores. However, current research tends to overlook such scores, instead focusing on links between hand tremors and shooter ability. For example, prior studies have demonstrated that elite air pistol shooters have lower hand tremor amplitudes than sub-elite air pistol shooters [1,5]. In addition, the complexity of hand tremors was significantly lower among the elite group participants compared to the sub-elite group [1]. Tremor complexity is quantified using sample entropy (SampEn) and multiscale entropy analysis (MSE) [9,14]. Such apparent discrepancies underscore the need for a better understanding of how hand tremor complexity impacts upper limb control in air pistol shooters. 

Muscle activation is one of the key factors influencing upper limb tremors. For example, Carignan et al. demonstrated that muscle activation can induce physiologic tremors [15], and Daneault et al. discovered that muscle co-contraction can induce physiologic tremors in the fingers [16]. Moreover, Novak et al. discovered that increased activation of some muscles has been linked to increased tremor amplitude [17]. In addition, upper limb muscle activation is associated with shooting performance in air pistol shooters. For example, Mon et al. discovered a linear correlation between shoulder abduction isometric force and shooting scores among elite male air pistol shooters [18]. Prior studies have investigated the relationship between muscle activation and shooting scores. Furthermore, it has been established that muscle activation can result in changed postural hand tremors. However, even though upper limb muscle activation is associated with physiologic tremors in healthy individuals, no studies currently exist that explore how upper limb muscle activation affects hand tremors in air pistol shooters during aiming.

The current study was designed to assess the relationship between hand tremors and the upper limb muscle activation of air pistol shooters during the aiming phase, which provides insights for training programs in order to reduce hand tremors. Therefore, to explore the underlying mechanisms affect hand tremors, the objectives of this study were (1) to examine the relationship between shooting scores and hand tremors when aiming, and (2) to explore the relationship between muscle activation and hand tremors when aiming. We hypothesized that (1) shooters with lower tremor amplitude and lower tremor complexity would have better shooting scores, and (2) shooters with increased muscle activation would experience more hand tremors during aiming.

## 2. Materials and Methods

### 2.1. Subjects

A total of 17 male 10 m air pistol shooters from China’s national team and the Air Pistol Sports Center participated in this study. The shooters were divided into two groups based on their training time and skill level, comprising an elite group (*n* = 10; age: 21.80 ± 6.63 years; years of experience: 7.33 ± 2.12 years) and a sub-elite group (*n* = 7; age: 18.00 ± 2.11 years; years of experience: 2.60 ± 0.98 years). Shooters in the elite group had over five years of air pistol training and had competed at the international or national level. Shooters in the sub-elite group had 1–3 years of air pistol training and were members of air pistol sports centers. The selection criteria were carried out according to Swann et al.’s study [19]. All participants were non-smokers, had no history of neurological disorders, and had refrained from consuming caffeinated products on the day of testing [20]. Written informed consent was obtained from all participants in accordance with the institutional review board.

### 2.2. Experiment Protocol

During the experiment, the shooters used their own equipment, as required by the International Shooting Sport Federation (ISSF) regulation, and this is a cross-sectional study. To assess hand tremors, a three-axis accelerometer (Xsens MTw Awinda, Enschede, The Netherlands; mass = 16 g; size = 4.7 × 3 × 1.3 cm; measurement accuracy = ±0.08 g (g = 9.8 m/s^2^); bandwidth = 180 Hz; sampling frequency = 150 Hz) was placed on the hand at the middle shaft of the third metacarpal bone [5]. The accelerometer measured acceleration data in the anteroposterior (AP), middle-lateral (ML), and vertical (VT) directions relative to limb segments (local axis). Surface electromyography (sEMG) of the right anterior deltoid, posterior deltoid, biceps brachii (short head), triceps brachii (long head), flexor carpi radialis, and extensor carpi radialis was recorded using the Delsys Trigno Avanti Sensor (Delsys Incorporated, Natick, MA, USA; sampling frequency = 2000 Hz) [21]. ACC data and sEMG data were synchronized for data collection. Following the placement of accelerometers and sEMG sensors, each shooter underwent a 5 to 10 min warm-up exercise (e.g., practice aiming and familiarizing himself with the target), as shooters usually do before competitions.

The experiments were conducted in a national-class competition shooting range specifically designed for air pistol shooters. Shooters fired their air pistols at a distance of 10 m from the target, aiming along the pistol barrel to simulate competitive conditions. Each shooter completed 40 shots, with accelerometer and sEMG data collected every 10 shots, totaling four rounds of data collection. Accelerometer and sEMG data were recorded continuously throughout the shooting process. After each shot, shooters could review their scores, which were displayed on an official ISSF-approved electronic target system, SUOOTER—ST10L (China). Scores were measured to one decimal place.

### 2.3. Data Processing

#### 2.3.1. Physiological Hand Tremor Data Processing

The initiation of each shot was identified by observing the accelerometer data time series from the hand in the vertical direction since the motion after each shot was most evident in this direction. Aiming time for air pistol shooters typically ranges from 5 to 10 s [12,13]. Accordingly, hand tremors for all three directions occurring within 5 s prior to a gunshot were analyzed. Linear trends within the accelerometer data were removed before assessing tremor strength.

The 40 shots taken by each participant in this study were analyzed. A second-order, zero-lag, low-pass Butterworth filter with a 50 Hz cut-off frequency was applied to the data for noise reduction [1,9]. The amplitude of hand tremors was analyzed using the RMS, which can determine tremor strength according to the following formula:

(1) $$RMS=\sqrt{\frac{\left(x_{1}^{2}+x_{2}^{2}+x_{3}^{2}\ldots x_{n}^{2}\right)}{n}}$$

**Figure 1:**
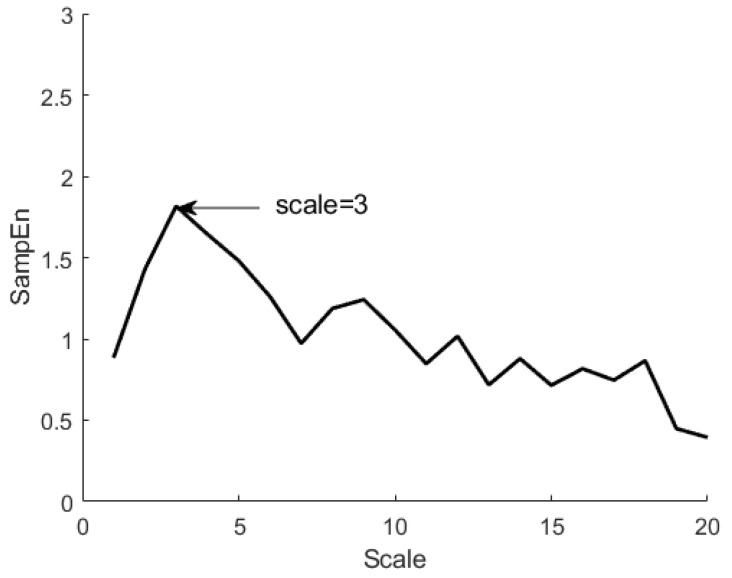
The multiscale entropy curve generated from the magnitude time series.

Tremor complexity was analyzed using both SampEn and MSE. MSE can address limitations of approximate entropy (ApEn) and SampEn, which have been widely used to investigate the complexity of human hand tremors [20,22]. SampEn was calculated for each coarse-grained time series as follows [9]:

(2) $$SampEn\left(m,r,N\right)=-ln\left[\frac{C^{m+1}(r)}{C^{m}}\right]$$

where m is the length of the repetition vector, r is the similarity criterion, N is the length of ACC data, and $C^{m}(r)$ is the correlation sum. In this study, m and r were set to 2 and 0.2, respectively [5]. Figure 1 shows the MSE curves generated by plotting sample entropy as a function of the timescale based on hand tremor data. A peak can be observed when the score equals 3. MSE was calculated as the area under the MSE curve as follows [1,14]:

(3) $$MSE=\sum_{i=1}^{3}sampEn(i)$$

The power of the tremor was assessed using power spectral density (PSD), which was measured in the AP, ML, and VT axes using Welch’s power spectral density estimate within the range of 1–40 Hz. Spectral analysis was performed using a 256 data-point-length fast Fourier transform (FFT, 128 data-point window size, 64 data-point overlap) [5,9]. For acceleration data, power from the dominant frequency peak (peak power) and the frequency at which the peak power occurred (Hz) were calculated from 6.3 to 12.5 Hz [10,23]. Peak power in PSD (PwrP) was used to indicate the peak power of the hand tremor [10]:

(4) $$Pwrp=\int_{f_{p}-0.5}^{f_{p}+0.5}\frac{1}{N^{2}}\left(S_{dft}^{*}\left(f\right)\times S_{dft}\left(f\right)\right)df$$

where $f_{p}$ denotes the bandwidth of the peak power of the hand tremor in the 8–12 Hz range [5]. Chenbin Ma et al. argue that the peak power metric is preferable to the one-side power spectrum of the sensor signal over a range of the dominant frequency ± 0.5 Hz around the length of the power estimate [10]. $S_{dft}^{*}\left(f\right)$ is the complex conjugate of $S_{dft}\left(f\right)$, and $S_{dft}\left(f\right)$ denotes the discrete Fourier transform of the expressed power signal.

#### 2.3.2. EMG Data Processing

With regard to EMG data processing, data for all six muscles were analyzed for five seconds before the gunshot. A fourth-order band-pass Butterworth filter with a 5–450 Hz cut-off frequency was applied to filter the EMG data, which was followed by a full-wave rectification. Subsequently, a fourth-order low-pass filter with a 20 Hz cut-off frequency was used to obtain the envelope. The maximum value of the envelope of each muscle during the 40 shots was determined to be a given shooter’s normalized EMG baseline. Muscle activation was then calculated via RMS.

### 2.4. Statistics

Statistical analyses were conducted using IBM SPSS Statistics 28.0.1.14 (Chicago, IL, USA). All data are presented as means and standard deviations across shooters and trials. The Shapiro–Wilk test was used to assess the normal distribution. Pearson correlation analysis was used to examine the relationship between the RMS, Pwrp, and MSE of the hand tremors in three directions and the scores of all shooters, as well as to examine the relationship between the RMS of the upper limb muscles and the RMS of the hand tremors of all shooters in three directions. r^2^ could be rated as weak (0 < r^2^ < 0.3), moderate (0.3 ≤ r^2^ < 0.5), high (0.5 ≤ r^2^ < 0.8), or strong (r^2^ ≥ 0.8). The dependent variables (RMS, Pwrp, MSE) for the elite and sub-elite groups were compared using independent *t*-tests, with effect size calculated according to Cohen’s method. Results were considered significant when the chance of making a Type 1 error was less than 5% (*p* < 0.05).

## 3. Results

**Figure 2:**
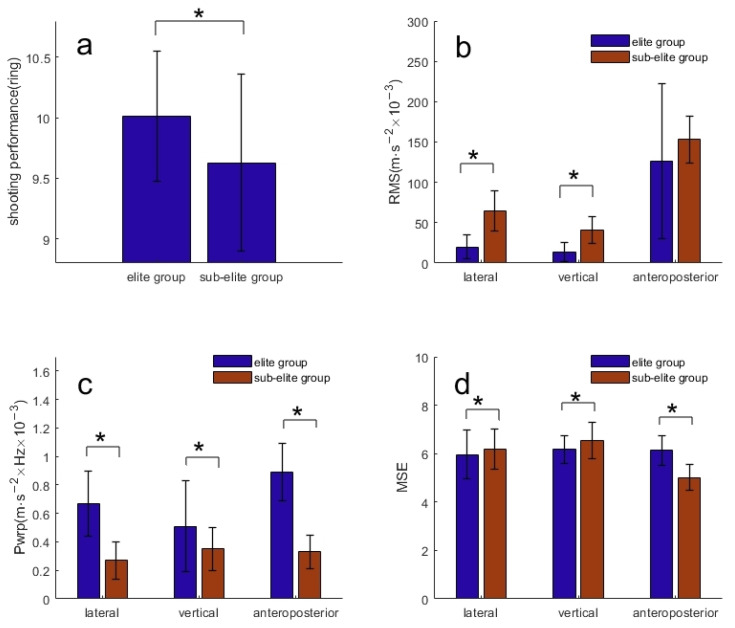
Scores of elite group and sub-elite group (**a**). The amplitude of hand tremors between the elite group and the sub-elite group (**b**). The peak powers of hand tremors between the elite group and the sub-elite group (**c**). The complexity of hand tremors between the elite group and the sub-elite group (**d**). * Significant tremor amplitude difference between elite group and sub-elite group: *p* < 0.05.

The mean shot score for the elite group was 10.01 ± 0.54, which was significantly higher than the sub-elite group (9.63 ± 0.73, *p* < 0.001; Figure 2a). Meanwhile, the sub-elite group exhibited significantly higher tremor amplitudes compared to the elite group in the ML and VT directions (*p* < 0.001; Figure 2b). In addition, the sub-elite group showed lower Pwrp but higher MSE in all directions compared to the elite group (*p* < 0.001).

For all shooters, significant but weak negative correlations were found between hand tremor amplitude when aiming and shooting scores in the ML and VT directions (Table 1). Tremor complexity also exhibited a significant but weak negative correlation with the shooting scores in the ML and VT directions (Table 1).

In the elite group, significant and moderate negative correlations were identified between hand tremor amplitude and shooting scores in the VT direction, as well as significant and weak negative correlations between hand tremor amplitude and shooting scores in the ML direction (Table 2). Among elite shooters, tremor complexity was found to be negatively correlated with shooting scores in the ML direction. However, in the sub-elite group, no significant correlations were found between tremor amplitude or tremor complexity and shooting scores (Table 2).

**Figure 3:**
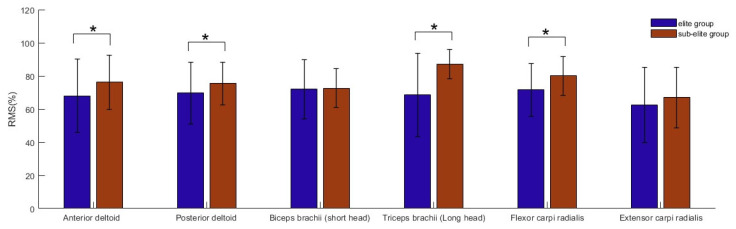
Normalized RMS of sEMG (% maximal EMG) in upper limb muscles between the elite group and the sub-elite group. * Significant tremor amplitude difference between elite group and sub-elite group: *p* < 0.05.

Furthermore, the sub-elite group participants demonstrated higher muscle activation in the anterior deltoid, posterior deltoid, triceps brachii (long head), and flexor carpi radialis compared with the elite group (*p* < 0.05, Figure 3).

For all shooters, significant and moderate positive correlations were found between the activation of the biceps brachii (short head) and hand tremors when aiming in the ML and VT directions (ML: r^2^ = 0.49, *p* = 0.010; VT: r^2^ = 0.44, *p* = 0.025, Table 3). For elite shooters, significant and moderate positive correlations were identified between the activation of the biceps brachii (short head) and hand tremors when aiming in the ML direction (ML: r^2^ = 0.49, *p* = 0.034, Table 4). However, in the sub-elite group, no significant relationship between upper limb activation and hand tremors was observed.

## 4. Discussion

Our findings revealed negative correlations between tremor amplitude, tremor complexity, and shooting scores among all shooters. In addition, positive correlations between tremor amplitude and muscle activation were identified within the elite group, but not in the sub-elite group.

A weak negative correlation between hand tremor amplitude (VL and AP directions) and shooting scores occurred in the elite group, which is consistent with our first hypothesis. This suggests that lower tremor amplitudes may contribute to higher shooting scores. Physiologic tremors consist of both centrally driven and mechanical-reflex components [10,24,25,26], and the central nervous system plays a crucial role in the modulation of muscle activation, especially in voluntary activation [15,16,25]. Some studies have proposed that muscle activation changes may impact upper limb stiffness, thereby affecting hand tremors [27,28]. From this, it can be assumed that sub-elite shooters with higher hand tremor amplitude exhibit greater central nervous system activity, leading to increased upper limb stiffness and reduced shooting scores. This finding aligns with Novak et al.’s study [17], which indicated that physiological tremor amplitude increases with higher neural drive to the muscles.

Another possible explanation for the link between decreased hand tremors and higher shooting scores may be related to the fact that elite shooters exert less attentive control over their hands when shooting [5]. When motor tasks require greater accuracy or are more complex (i.e., goal-direction position in air pistol shooting), hand tremor amplitude tends to increase [5]. Sub-elite shooters may focus more on visuomotor information during aiming, whereas elite shooters may possess a more natural aiming position which allows them to focus on the target rather than the need to maintain their aiming position, leading to higher shooting scores [5]. This familiarity with and increased focus on the target may help to increase upper limb stability, improving air pistol shooting accuracy.

In the elite group, weak negative correlations between tremor complexity (VT and AP directions) and shooting scores were identified, which is consistent with our first hypothesis. This also aligns with findings from Ko et al., who showed that higher shooting scores were related to a decrease in the complexity of hand tremors among experienced shooters during the aiming phase compared with less experienced shooters [20]. Lower hand tremor complexity may result from long-term shooting practice, which can improve upper limb control and coordination [1,20]. Air pistol shooters with greater hand tremor complexity might be said to possess greater exploratory behavior, meaning those movements made by human beings or other animals when orienting to new environments. In the context of air pistol shooting, exploratory behavior refers to exploring postures to maintain upper limb control during aiming [1]. From this, it can be concluded that sub-elite shooters’ deficiency in aiming may occur because they are still seeking a posture to achieve accuracy. Similarly, Zhou et al.’s study found that low tremor complexity leads to higher stress tolerance and improved shooting scores [29]. Differences in hand tremor complexity could also be attributed to sub-elite shooters’ need for a greater degree of freedom (DOF) to control upper limb movement when aiming, while elite shooters are able to maintain stability through a more direct connection between their shoulder and wrist joints [20]. Having a greater DOF in the upper limbs presents a challenge for sub-elite shooters when attempting to maintain stability, which may lead to more complex tremors and lower shooting scores. Therefore, the ability to unconsciously achieve an effective posture while aiming may improve upper limb stability as well as shooting scores for air pistol shooters.

In the sub-elite group, no correlation was observed between hand tremors and shooting scores. The reason for this may stem from a multitude of factors contributing to errors among sub-elite shooters, which extend beyond hand tremors to include aspects such as aiming time and aiming accuracy [12,13]. While both elite and sub-elite shooters are able to attain a score of 10, elite shooters demonstrate consistent performance at this level, whereas sub-elite shooters frequently score in the 8–9 range. This is the reason why correlations appeared between shooting scores and hand tremors in the elite shooters but not the sub-elite shooters; for sub-elite shooters, shooting performance consistency is prioritized over the occasional high score.

The sub-elite shooters also exhibited greater muscle activation compared to the elite shooters, which can likely be attributed to a shorter training duration. Consequently, sub-elite shooters may constantly contract their muscles during aiming to adjust their position, resulting in higher muscle activation [5,23]. Additionally, the study of Carignan et al. indicated that hand tremors are mainly generated by the angular movement of the shoulder joint [25]. Anterior deltoid, posterior deltoid, and triceps brachii (long head) contractions can change the angular movement of the shoulder joint. Therefore, differences in the upper limb muscle activation of elite and sub-elite air pistol shooters may lead to differences in shooting postures, resulting in different types of hand tremors.

In the elite group, weak positive correlations were found between biceps brachii (short head) activation and hand tremors in the ML and VT directions, which is consistent with our second hypothesis. Biceps brachii (short head) can act as an adductor, facilitating movement of the humerus towards the body’s midline [30]. As the activation of this muscle increases, hand tremors increase as well. However, this does not affect the activation of the triceps brachii (long head). Lakie et al.’s study showed that instability between the agonist and antagonist muscle groups can exacerbate hand tremors [27]. Thus, the higher number of hand tremors in the ML direction for elite shooters might be due to an imbalance in the activation of shoulder adduction and abduction muscles. This suggests that elite air pistol shooters could attempt to balance the activation of adduction and abduction muscles by increasing the strength of their abduction muscles, such as the triceps brachii (long head), to reduce hand tremors and improve shooting scores. The results of this study demonstrated no significant associations between activation of the anterior deltoid, posterior deltoid, flexor carpi radialis, and extensor carpi radialis and hand tremors in air pistol shooters. This suggests that among air pistol shooters, hand tremors may not only be determined by muscle activation in and around the hand but also by muscle activation around the shoulder.

## 5. Conclusions

This study identified negative correlations between shooting scores and hand tremors among all shooters who participated, indicating that a decrease in hand tremors is associated with better shooting scores, particularly among elite shooters. Furthermore, our findings showed that higher muscle activation of the triceps brachii (long head) is correlated with higher hand tremor amplitude in elite shooters. This suggests that reducing the activation of the biceps brachii (long head) or increasing the activation of the triceps brachii, that is, stabilizing both the agonist and antagonist muscles, might be an effective method for reducing hand tremors. By balancing the agonist and antagonist muscles of the shoulder joint, shooters can potentially reduce hand tremors and improve their shooting sores.

## Figures and Tables

**Table 1:** The relationships between tremor parameters and shooting scores for all shooters.

Parameter		r^2^	*p*-Value
RMS	ML	−0.22 *	<0.001
	VT	−0.25 *	<0.001
	AP	−0.01	0.769
Pwrp	ML	−0.26 *	<0.001
	VT	−0.30 *	<0.001
	AP	−0.18	0.216
MSE	ML	−0.26 *	<0.001
	VT	−0.28 *	<0.001
	AP	−0.05	0.205

* represents that the correlation was statistically significant.

**Table 2:** The relationship between tremor parameters and shooting scores for the elite and sub-elite groups.

Parameter		Elite Group (*n* = 10)		Sub-Elite Group (*n* = 7)	
		r^2^	*p*-Value	r^2^	*p*-Value
RMS	ML	−0.27 *	<0.001	−0.05	0.311
	VT	−0.33 *	<0.001	0.10	0.172
	AP	−0.04	0.472	−0.06	0.590
Pwrp	ML	−0.10	0.252	−0.08	0.426
	VT	−0.01	0.209	−0.16	0.844
	AP	−0.11	0.285	−0.12	0.165
MSE	ML	−0.31 *	<0.001	0.02	0.782
	VT	−0.08	0.922	0.05	0.254
	AP	−0.08	0.591	0.06	0.517

* represents that the correlation was statistically significant.

**Table 3:** Relationship between root mean square (RMS) of hand tremor amplitude and RMS of EMG (*n* = 17).

Muscle Name	ML		VT		AP	
	r^2^	*p*-Value	r^2^	*p*-Value	r^2^	*p*-Value
Anterior deltoid	−0.05	0.800	0.07	0.726	−0.03	0.897
Posterior deltoid	0.16	0.446	−0.01	0.946	0.13	0.530
Biceps brachii (short head)	0.49 *	0.010	0.44 *	0.025	0.27	0.183
Triceps brachii (long head)	−0.13	0.524	−0.05	0.813	−0.32	0.110
Flexor carpi radialis	0.02	0.942	0.09	0.651	0.15	0.463
Extensor carpi radialis	0.24	0.231	0.25	0.227	0.21	0.314

* represents that the correlation was statistically significant.

**Table 4:** The relationship between RMS of hand tremor amplitude and RMS of EMG in elite group (*n* = 10).

Muscle Name	ML		VT		AP	
	r^2^	*p*-Value	r^2^	*p*-Value	r^2^	*p*-Value
Anterior deltoid	0.09	0.719	0.17	0.494	0.12	0.636
Posterior deltoid	0.32	0.181	0.09	0.766	0.28	0.245
Biceps brachii (short head)	0.49 *	0.034	−0.39	0.357	0.17	0.476
Triceps brachii (long head)	0.05	0.826	0.22	0.103	−0.18	0.455
Flexor carpi radialis	−0.02	0.954	0.09	0.686	−0.12	0.629
Extensor carpi radialis	−0.11	0.677	−0.25	0.312	−0.07	0.766

* represents that the correlation was statistically significant.

## Data Availability

Data are contained within the article.
